# Differential Regulation of Extracellular Matrix and Soluble Fibulin-1 Levels by TGF-β_1_ in Airway Smooth Muscle Cells

**DOI:** 10.1371/journal.pone.0065544

**Published:** 2013-06-07

**Authors:** Ling Chen, Qi Ge, Judith L. Black, Linhong Deng, Janette K. Burgess, Brian G. G. Oliver

**Affiliations:** 1 Key Laboratory of Biorheological Science and Technology, Ministry of Education, Bioengineering College, Chongqing University, Shapingba, Chongqing, China; 2 Woolcock Institute of Medical Research, Sydney, New South Wales, Australia; 3 Discipline of Pharmacology, The University of Sydney, Sydney, New South Wales, Australia; 4 Institute of Biomedical Engineering and Health Sciences, Changzhou University, Changzhou, Jiangsu, China; The University of Hong Kong, Hong Kong

## Abstract

Fibulin-1 (FBLN-1) is a secreted glycoprotein that is associated with extracellular matrix (ECM) formation and rebuilding. Abnormal and exaggerated deposition of ECM proteins is a hallmark of many fibrotic diseases, such as chronic obstructive pulmonary disease (COPD) where small airway fibrosis occurs. The aim of this study was to investigate the regulation of FBLN-1 by transforming growth factor beta 1 (TGF-β_1_) (a pro-fibrotic stimulus) in primary human airway smooth muscle (ASM) cells from volunteers with and without COPD. Human ASM cells were seeded at a density of 1×10^4^ cells/cm^2^, and stimulated with or without TGF-β_1_ (10 ng/ml) for 72 hours before FBLN-1 deposition and soluble FBLN-1 were measured. Fold change in FBLN-1 mRNA was measured at 4, 8, 24, 48, 72 hours. In some experiments, cycloheximide (0.5 µg/ml) was used to assess the regulation of FBLN-1 production. TGF-β_1_ decreased the amount of soluble FBLN-1 both from COPD and non-COPD ASM cells. In contrast, the deposition of FBLN-1 into the ECM was increased in ASM cells obtained from both groups. TGF-β_1_ did not increase FBLN-1 gene expression at any of the time points. There were no differences in the TGF-β_1_ induced FBLN-1 levels between cells from people with or without COPD. Cycloheximide treatment, which inhibits protein synthesis, decreased both the constitutive release of soluble FBLN-1, and TGF-β_1_ induced ECM FBLN-1 deposition. Furthermore, in cycloheximide treated cells addition of soluble FBLN-1 resulted in incorporation of FBLN-1 into the ECM. Therefore the increased deposition of FBLN-1 by ASM cells into the ECM following treatment with TGF-β_1_ is likely due to incorporation of soluble FBLN-1 rather than de-novo synthesis.

## Introduction

Chronic obstructive pulmonary disease (COPD), a common preventable and treatable disease, is characterised by airflow limitation that is usually progressive and associated with an enhanced chronic inflammatory response in the airways and the lung to noxious particles or gases [1]. It is known that the extracellular matrix (ECM) is altered in the airway walls of patients with COPD [2], [3]. Increased airway thickening and narrowing occurs in the small airways of smokers with COPD, which is the main reason for respiratory obstruction [4], [5], [6], [7].

The ECM is a complex structured network of secreted macromolecules and proteolytic enzymes that provide the basis of cell-cell and cell-matrix interactions. In the lung, ECM components fundamentally influence the structure and function of airways. Abnormal and exaggerated deposition of the ECM is a hallmark of many fibrotic diseases, such as COPD. Fibulin-1 (FBLN-1) is a secreted glycoprotein that is associated with ECM formation and rebuilding. FBLN-1 is expressed in basement membranes, microfibrils and elastic fibres [8], and also is associated with various ECM proteins such as fibronectin (FN), nidogen-1, and laminin-1 [9], [10], [11], [12]. However, the role of FBLN-1 in the aetiology and pathology of fibrosis is unclear. We have previously found elevated FBLN-1 in the serum and lung washing fluid (bronchoalveolar lavage) of people with asthma, and furthermore shown FBLN-1 to regulate airway smooth muscle (ASM) cell proliferation, therefore highlighting the potential role of FBLN-1 in airway wall remodelling [13].

Transforming growth factor beta 1 (TGF-β_1_) is a pro-fibrotic cytokine which is increased in several forms of acute and chronic adult lung diseases such as asthma [14], COPD [15], [16], and pulmonary fibrosis [17], [18]. It is considered to play a crucial role in the pathogenesis of tissue fibrosis, stimulating the production of various collagens and ECM proteins [19], [20], [21]. The regulation of ECM production by TGF is often different between primary mesenchymal lung cells from people with fibrotic lung diseases in comparison to those without. For example we have previously found that TGF-β_1_ increased perlecan from COPD ASM cells only [22].

This study aimed to investigate the regulation of FBLN-1 by TGF-β_1_ in primary human ASM cells from volunteers with or without COPD. We hypothesized that the pro-fibrotic cytokine TGF-β_1_ would up regulate the deposition of FBLN-1, with greater production observed in cells from people with COPD incomparison to cells from people without lung disease.

## Materials and Methods

### Ethics Statement

Approval of all experiments with human lung tissues was provided by the Ethics Review Committee of the South West Sydney Area Health Service, St Vincent’s Hospital Sydney, Strathfield Private Hospital, Royal Prince Alfred Hospital, and the University of Sydney Human Research Ethics Committee. All volunteers or their next of kin provided written informed consent.

### Study Population

Samples obtained from a total of 17 volunteers with COPD and 19 volunteers without COPD were studied. COPD was diagnosed according to current guidelines including dyspnea, chronic cough or sputum production, a history of exposure to risk factors for the disease, and spirometry [1]. Those in the COPD group had a forced expiratory volume in one second (FEV_1_)/forced vital capacity (FVC) ≤0.7 indicating airflow limitation. The non-COPD group had a FEV_1_/FVC >0.7 and FEV_1_≥80%. The details of all individuals from whom tissue was obtained are provided in table 1.

**Table 1:** Characteristics of volunteers.

	Surgery	Diagnosis	Age, yr	Gender	Smoker	FEV_1_:FVC	FEV_1%_	FVC %
1	T	Emphysema	54	M	Y	0.26	14	44
2	T	Emphysema	53	M	Y	0.34	23	55
3	T	Emphysema	51	F	Y	0.27	22	67
4	T	Emphysema	45	F	Y	0.24	21	69
5	T	Emphysema	67	M	Y	0.24	13	44
6	T	Emphysema	57	F	Y	0.33	24	59
7	T	Emphysema	51	F	Y	0.25	18	57
8	T	Emphysema	60	M	Y	0.25	9	30
9	R	COPD	59	M	Y	0.74	69	71
10	R	COPD/NSCCa	64	M	Y	0.60	56	73
11	R	COPD/NSCCa	58	M	Y	N/A	70	N/A
12	R	COPD/SCCa	71	M	Y	0.55	44	64
13	R	NSCCa	60	M	Y	0.60	77	97
14	R	NSCCa	72	M	Y	0.74	51	53
15	R	Adeno Ca	56	F	Y	0.74	66	71
16	R	Neoplasm	68	F	Y	0.67	72	81
17	T	Sarcoidosis	43	M	Y	0.55	31	45
18	B	Normal	22	F	Y	0.94	86	91
19	B	Normal	29	M	N	0.84	86	84
20	B	Normal	22	F	N/A	N/A	N/A	N/A
21	B	Normal	27	F	N	N/A	N/A	N/A
22	T	Normal	16	M	N/A	N/A	N/A	N/A
23	B	Normal	69	M	N/A	N/A	N/A	N/A
24	N/A	Normal	27	M	N	N/A	N/A	N/A
25	R	NSCCa	72	M	Y	0.72	83	89
26	R	NSCCa	63	M	Y	0.85	68	64
27	R	NSCCa	59	M	Y	0.78	75	73
28	R	NSCCa	73	F	Y	0.74	100	113
29	R	NSCCa	70	F	Y	0.68	64	92
30	R	NSCCa	65	M	Y	0.78	92	93
31	R	NSCLC	70	M	Y	0.73	85	88
32	R	Ca	78	F	Y	0.76	88	95
33	R	Ca	61	M	Y	0.75	101	107
34	T	IPF	55	M	Y	0.88	39	35
35	T	IPF	58	M	Y	0.88	52	44
36	T	IPF	65	M	Y	0.74	30	32

T, transplant; R, resection; B, bronchoscopy; M, male; F, female; Y, yes; N, no; N/A, not available; FEV_1_%, forced expiratory volume in 1 second of predicted %; FVC%, forced vital capacity of predicted %; Ca, carcinoma; SCCa, small cell carcinoma; NSCCa, non-small cell carcinoma; NSCLC: non-small cell lung carcinoma; IPF: idiopathic pulmonary fibrosis.

### Isolation of Human ASM Cells

Human ASM cells were isolated from lung tissue obtained from donors undergoing resection for either thoracotomy or transplantation. Methods for isolation of the cells were described previously [23]. In short, bronchial airways were dissected from the surrounding lung tissue and cut longitudinally. Subsequently, the airways were washed in 80% ethanol and Hank’s balanced salt solution (Invitrogen, Carlsbad, CA, USA) before being dissected under a dissecting microscope. Isolated smooth muscle bundles were cut into pieces and placed into 25 cm^2^ tissue culture flasks (BD Biosciences, North Ryde, Australia) containing 2.5 ml Dulbecco’s modified eagle’s medium (DMEM) (Invitrogen) supplemented with 10% foetal bovine serum (FBS) (DKSH, Melbourne, AUS), 2% antibiotics (Invitrogen), 25 mM Hepes (Invitrogen) and 5 µg/ml plasmocin (InvivoGen, San Digo, CA, USA) and placed in a humidified CO_2_ incubator (37°C/5% CO_2_). Twice a week, the medium was aspirated and replaced with fresh DMEM supplemented with 5% FBS, 1% antibiotics and 25 mM Hepes (growth medium). Cells used for experiments between passages 2 and 7, and all the cells tested negative for the presence of mycoplasma before they were set up for experiments.

### ASM Cell Culture

ASM cells were seeded in 6-well plates and 96-well plates at a density of 1×10^4^ cells/cm^2^ in growth medium and incubated at 37°C/5% CO_2_. After 72 hours, cells were quiesced in DMEM supplemented with 0.1% FBS, 1% antibiotics and 25 mM Hepes (Quiescing medium) for 24 hours then treated with or without 10 ng/ml of TGF-β_1_ (R&D Systems, Minneapolis, MN, USA) in quiescing medium for 4, 8, 24, 48 (RNA analysis) and 72 hours (RNA and protein analysis). RNA samples were stored at −80°C, and supernatant and protein samples were stored at −20°C until analysis.

### RNA Extraction and Real Time Polymerase Chain Reaction (PCR)

Total RNA was collected over a time course (4, 8, 24, 48, 72 hours) using the ISOLATE RNA Mini kit (Bioline, London, UK) according to the manufacturer’s instructions. RNA was eluted in 50 µl of RNase free water. mRNA was converted to cDNA by using M-MLV reverse transcriptase (Invitrogen) according to the manufacturer’s instructions. For the detection of FBLN-1 and FN gene expression the primers were used as follows: FBLN-1 Hs00243545_ml and FN Hs00365058_ml (Invitrogen) [13]. For quantitative analysis of gene expression, human 18S rRNA (Invitrogen) was used as an endogenous control. The thermal cycle conditions consisted of 40 cycles of 95°C for 15 seconds and 60°C for 1 minute [24]. Real time PCR was performed using the StepOne Plus detection system, data were collected and analysed by StepOne software (Applied Biosystems, Melbourne, AUS). The relative abundance of mRNA was calculated by using the ΔΔCt method [25], and results were normalized to 18S rRNA.

### ECM Enzyme-linked Immune Sorbent Assay (ELISA)

Human ASM cells were seeded in 96-well plates as described above. After stimulation with or without 10 ng/ml TGF-β_1_ for 72 hours, ASM cells were lyzed with 0.016 mM NH_2_OH at 37°C for 20 minutes. The cell free ECM plates were washed three times with 0.05% phosphate buffered saline (PBS)-Tween (vol/vol) and stored with 100 ul/well of PBS at −20°C until analysis. The deposition of protein in the ECM was measured by ELISA according to the method described previously [26]. Briefly, ECM plates were defrosted at room temperature, and non-specific bound molecules were removed by blocking with 1% BSA in PBS (Sigma Aldrich, St Louis, MO, USA). All antibodies were diluted in 1% BSA/PBS-Tw and added at 50 µl/well. Mouse monoclonal anti-human FBLN-1 antibody (0.07 µg/ml) (Santa Cruz Biotechnology, Santa Cruz, CA, USA) and mouse monoclonal anti-human FN C-terminal antibody (2 µg/ml) (Millipore, Billerica, MA, USA) were used as primary antibodies. Mouse IgG1,κ (BD Biosciences, Franklin Lakes, NJ, USA) and mouse IgG2a (Dako, Glostrup, DK) were used as isotype controls respectively. For measurement of FBLN-1 or its isotype control, a biotinylated chicken anti-mouse antibody (8 µg/ml) (Santa Cruz) was used followed by streptavidin-horseradish peroxidase (streptavidin-HRP) (1∶200 dilution) (R&D Systems). Rabbit anti-mouse Ig-HRP (2.6 µg/ml) (Dako) was used as a secondary antibody for the measurement of FN and its isotype control. After final washing, 50 µl/well of chromogenic substrate 3,3′,5,5′-tetramethylbenzidine (TMB) was added, the reaction was stopped by 50 µl/well of 1M N_2_HPO_3_ and absorbance was read at 450 nm–570 nm using a spectrophotometer and software (Spectramax M2 and Soft Max pro 4.8, Molecular Devices, Sunnyvale, CA, USA).

### Protein Extraction and Western Blots

Human ASM cells were seeded in 6-well plates as described above, after stimulation with or without 10 ng/ml TGF-β_1_, supernatant and total cellular protein were collected and stored at −20°C until analysis. Total cellular protein was extracted by using extracting buffer which contained 20 mM Tris, pH 7.4, 150 mM NaCl, 1 mM Na_2_EDTA, 1% Triton X-100, 10% glycerol, 0.1% SDS, 0.5% sodium deoxycholate, 1% protease inhibitor cocktail set III (Millipore) and 1 mM phenylmethylsulfonyl fluoride (PMSF) (Amresco, Solon, OH, USA). Cell lysates were collected and centrifuged at 4°C/14,000 g for 10 minutes to pellet cell debris. Total protein concentration was measured by Bio-Rad protein assay (Bio-Rad, Hercules, CA) according to the manufacturer’s instructions. Soluble FBLN-1 or FN and cellular FBLN-1 or FN were detected by western blots which were performed as described previously [27]. Proteins were size fractionated on 10% polyacrylamide gels, transferred to polyvinylidene fluoride (PVDF) membranes, and blocked in 5% (wt/vol) skim milk solution for 1 hour. The membranes were incubated with primary antibody (0.4 µg/ml of mouse monoclonal anti-human FBLN-1 antibody in 2% BSA/TBS-Tw) for 2 hours, followed by incubation with secondary antibody (2.6 μ/ml of rabbit anti-mouse Ig-HRP antibody in 2% BSA/TBS-Tw) for 1 hour. Immunoblot detection was performed using Immobilon Western Chemiluminescent HRP Substrate (Millipore) and bands were analysed by using Kodak image station 4000 MM, and the amount of protein present in each sample was determined as the densitometric density. Glyceraldehyde 3-phophate dehydrogenase (GAPDH) was used as a loading control, the amount of FBLN-1 or FN was normalized to GPADH detected on the same membrane, and results were expressed as fold change relative to control.

### Inhibition of New Protein Synthesis

Cycloheximide treatment was performed as described previously [28]. In short, human ASM cells were treated with 0.5 µg/ml cycloheximide or vehicle (DMSO) for 1 hour. Medium was aspirated from each well then cells were treated with or without 10 ng/ml TGF-β_1_ in the presence of cycloheximide or vehicle. After 72 hours, supernatant and cell free ECM plates were harvested and stored at −20°C for assessment of ECM proteins. Separately, another set of cells was counted for estimation of total number.

### Addition of Soluble FBLN-1

Supernatant from human ASM cells treated with quiescing medium for 72 hours was used as a source of soluble FBLN-1 containing medium. Human ASM cells were seeded in 96-well plates, grown and quiescenced as described before. Cells were pre-treated with 0.5 µg/ml cycloheximide for 1 hour then the medium was changed to a quiescing one or soluble FBLN-1 containing medium in the presence of cycloheximide with or without 10 ng/ml TGF-β_1_. After 72 hours, the cell free ECM plates were harvested and stored at −20°C for assessment of ECM proteins.

### Statistical Analysis

Data analysis was performed using GraphPad Prism 5.0 software (GraphPad Software, San Diego, CA, USA). All the data were expressed as mean ± standard error of the mean (SEM). Statistical significance was determined by paired t-test, one-way analysis of variance (one-way ANOVA) or two-way analysis of variance (two-way ANOVA) where appropriate, followed by Bonferroni post test. A p value of less than or equal to 0.05 (p≤0.05) was considered significant.

## Results

### TGF-β_1_ Up Regulated the Deposition of FBLN-1 into the ECM Produced by Both COPD and Non-COPD ASM Cells

**Figure 1:**
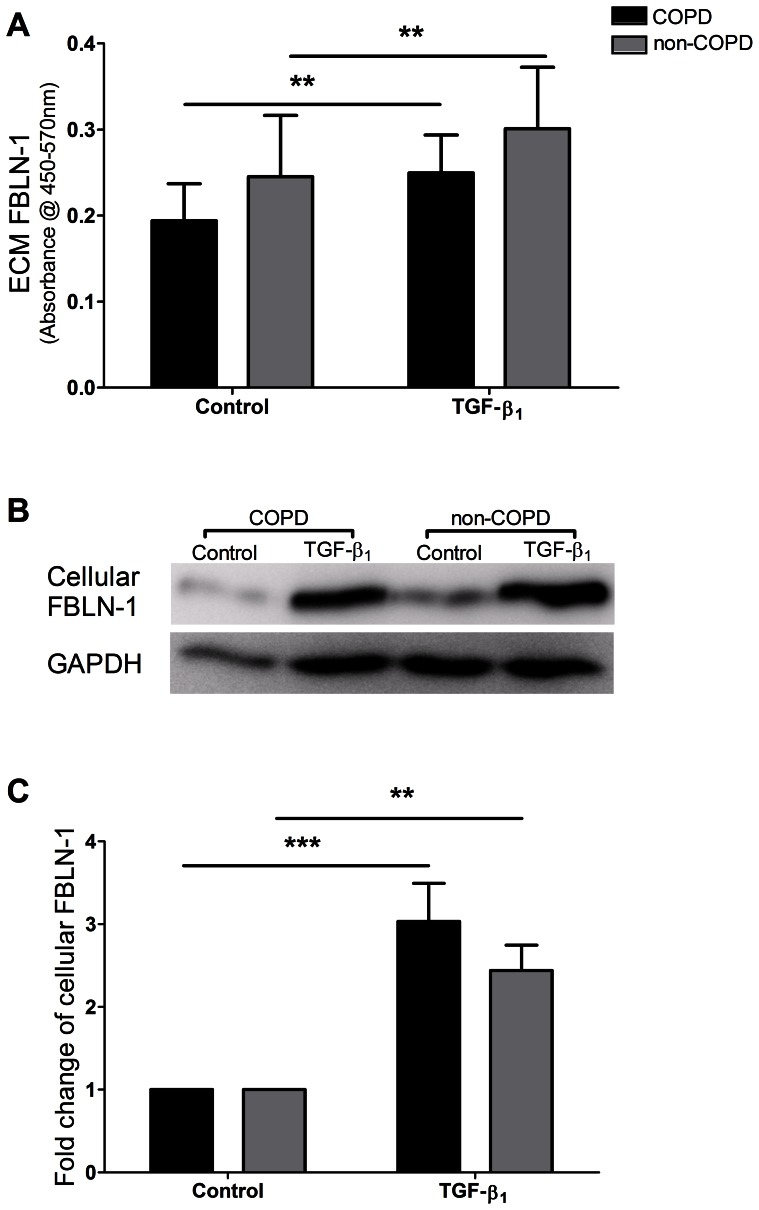
TGF-β_1_ increased the deposited and cellular FBLN-1 in human ASM cells. After 72 hours stimulation with 10 ng/ml TGF-β_1_, the deposition of FBLN-1 by human ASM cells obtained from COPD (black bar, n = 10) and non-COPD (grey bar, n = 7) was measured by ELISA and data were expressed as absorbance at 450 nm–570 nm (panel A). Cellular FBLN-1 and GAPDH from COPD and non-COPD ASM cells were detected by western blot (panel B). Data from COPD (black bar, n = 8) and non-COPD (grey bar, n = 7) group were normalized to GAPDH and expressed as fold change relative to control (panel C). Data were expressed as mean ± SEM, two-way ANOVA with Bonferroni post tests, **P<0.01, ***P<0.001, compared with control.

Following 72 hours stimulation with 10 ng/ml TGF-β_1_, the deposition of FBLN-1 was significantly increased in ASM cells obtained from volunteers with COPD (p<0.01, n = 10) and without COPD (p<0.01, n = 7) (Fig. 1A). TGF-β_1_ also increased the deposition of FN in ASM cells from both groups (COPD, p<0.01, n = 12 and non-COPD, p<0.05, n = 11) (Fig. S1A). There were no differences between COPD and non-COPD ASM cells in the deposition of FBLN-1 or FN either basally or after treatment with TGF-β_1_ (Fig. 1A, Fig. E1A).

### TGF-β_1_ up Regulated Cellular FBLN-1 in Both COPD and Non-COPD ASM Cells

As the deposition of FBLN-1 into the ECM was increased by TGF-β_1_, cellular FBLN-1 was assessed by western blotting to confirm this up regulation. After 72 hours of stimulation with 10 ng/ml TGF-β_1_, the FBLN-1 was significantly up regulated in both COPD (p<0.001, n = 8) and non-COPD (p<0.05, n = 7) ASM cells, and there was no difference between the COPD and non-COPD groups in the fold change of cellular FBLN-1 induced by TGF-β_1_ (Fig. 1B, 1C). The amount of cellular FN was increased by TGF-β_1_ in human ASM cells (p<0.05, n = 6) (Fig. S1B, S1C).

### TGF-β_1_ Decreased Soluble FBLN-1 Levels in Supernatants from COPD and Non-COPD ASM Cells

**Figure 2:**
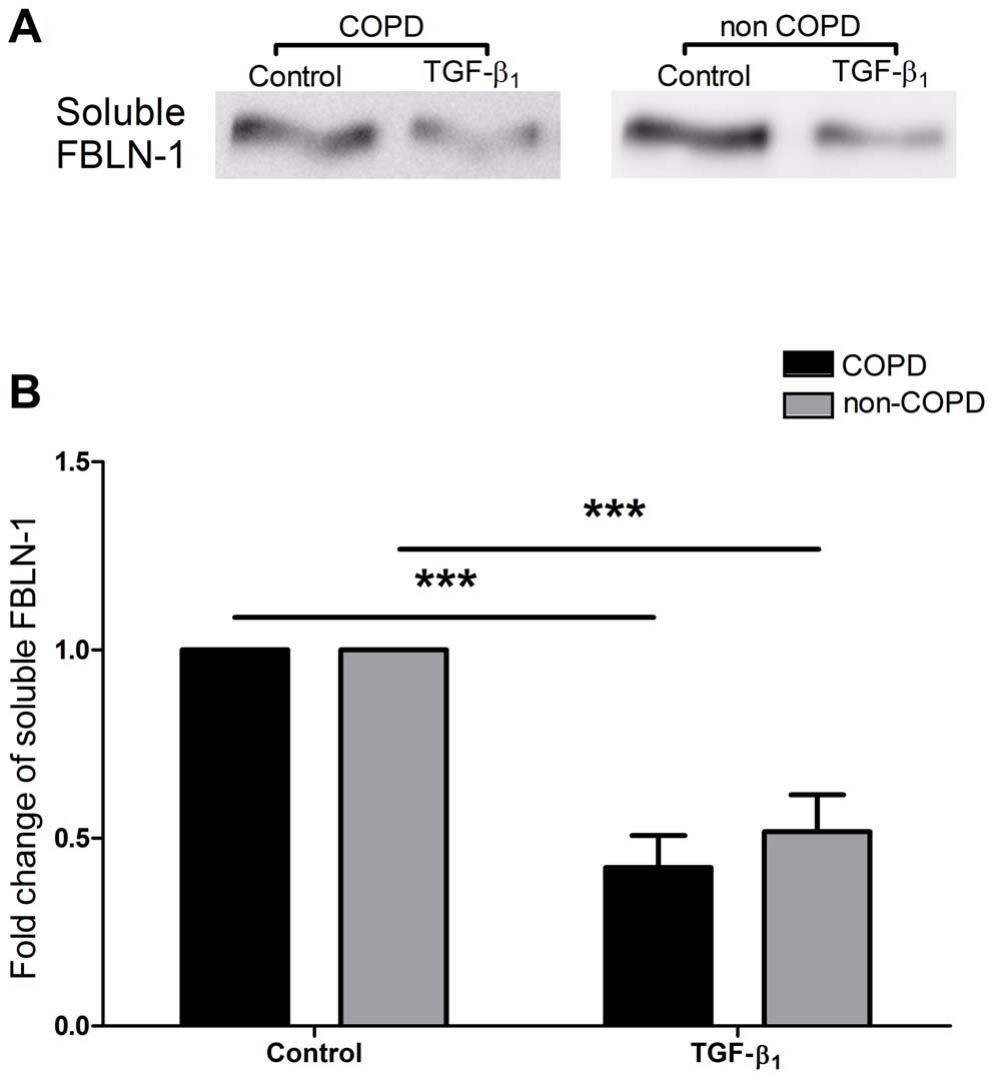
TGF-β_1_ decreased soluble FBLN-1 from human ASM cells. Soluble FBLN-1 released from human ASM cells was detected by western blot, following 72 hours stimulation with 10 ng/ml TGF-β_1_ (panel A). Data from COPD (black bar, n = 9) group and non-COPD (grey bar, n = 9) group were expressed as fold change relative to control (panel B). Data were expressed as mean ± SEM and analysed by two-way ANOVA with Bonferroni post tests, ***P<0.001, compared with control.

Since it’s already known that FBLN-1 exists in both solid (ECM) and soluble forms, we also measured soluble FBLN-1 in supernatants from human ASM cells. Following 72 hours stimulation with 10 ng/ml TGF-β_1_, the level of soluble FBLN-1 was significantly decreased in supernatant from COPD (p<0.001, n = 9) and non-COPD (p<0.001, n = 9) ASM cells, and there was on differences in the fold change of soluble FBLN-1 decreased by TGF-β_1_ between the two groups (Fig. 2A, 2B). In contrast, the level of soluble FN was significantly increased in the supernatants from human ASM cells by TGF-β_1_ (p<0.05, n = 8) (Fig. S2A, S2B).

### Reduction of FBLN-1 mRNA Expression by TGF-β_1_

**Figure 3:**
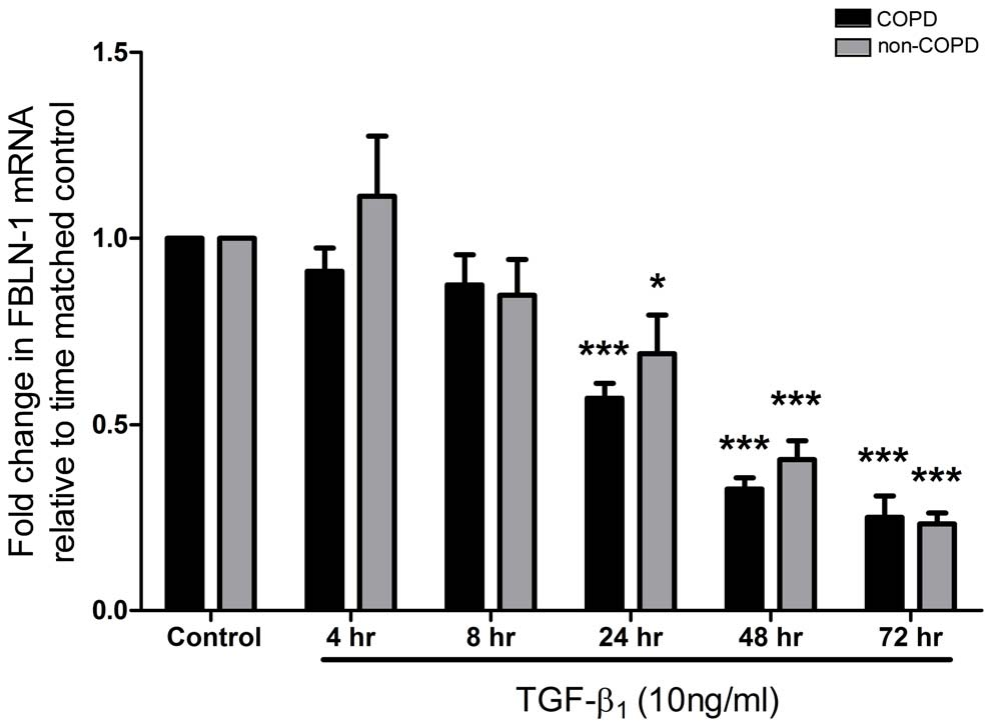
TGF-β_1_ decreased FBLN-1 gene expression in human ASM cells. COPD (black bar, n = 7) and non-COPD (grey bar, n = 5) human ASM cells were stimulated with 10 ng/ml TGF-β_1_, and FBLN-1 mRNA expression was detected during the time course by real time PCR. Results were normalized to the endogenous control (18S rRNA), and expressed as fold change in FBLN-1 mRNA compared with time matched control. Data were expressed as mean ± SEM and analysed by two-way ANOVA with Bonferroni post tests, *P<0.01, ***P<0.001, compared with time matched control.

After stimulation with 10 ng/ml TGF-β_1_, FBLN-1 mRNA expression in human ASM cells obtained from volunteers with and without COPD gradually decreased over the time course (4, 8, 24, 48, 72 hours) (Fig. 3). At the 4 h and 8 h time points there were no significant changes in FBLN-1 mRNA levels, however, at 24 h, 48 h, and 72 h time points the FBLN-1 mRNA expression level was significantly down regulated by TGF-β_1_ both in COPD (p<0.001, n = 7) and non-COPD (p<0.05, p<0.001, n = 5) ASM cells. In contrast, FN mRNA expression gradually increased following stimulation with TGF-β_1_ over the time course with significant increases at 48 h and 72 h time points in COPD (p<0.01, n = 7) ASM cells, and significant increases at 24 h, 48 h, and 72 h time points in non-COPD (p<0.01, n = 5) ASM cells (Fig. S3). The changes in FBLN-1 or FN mRNA were not different between the COPD and non-COPD groups.

### Cycloheximide Inhibits New FBLN-1 Protein Synthesis

**Figure 4:**
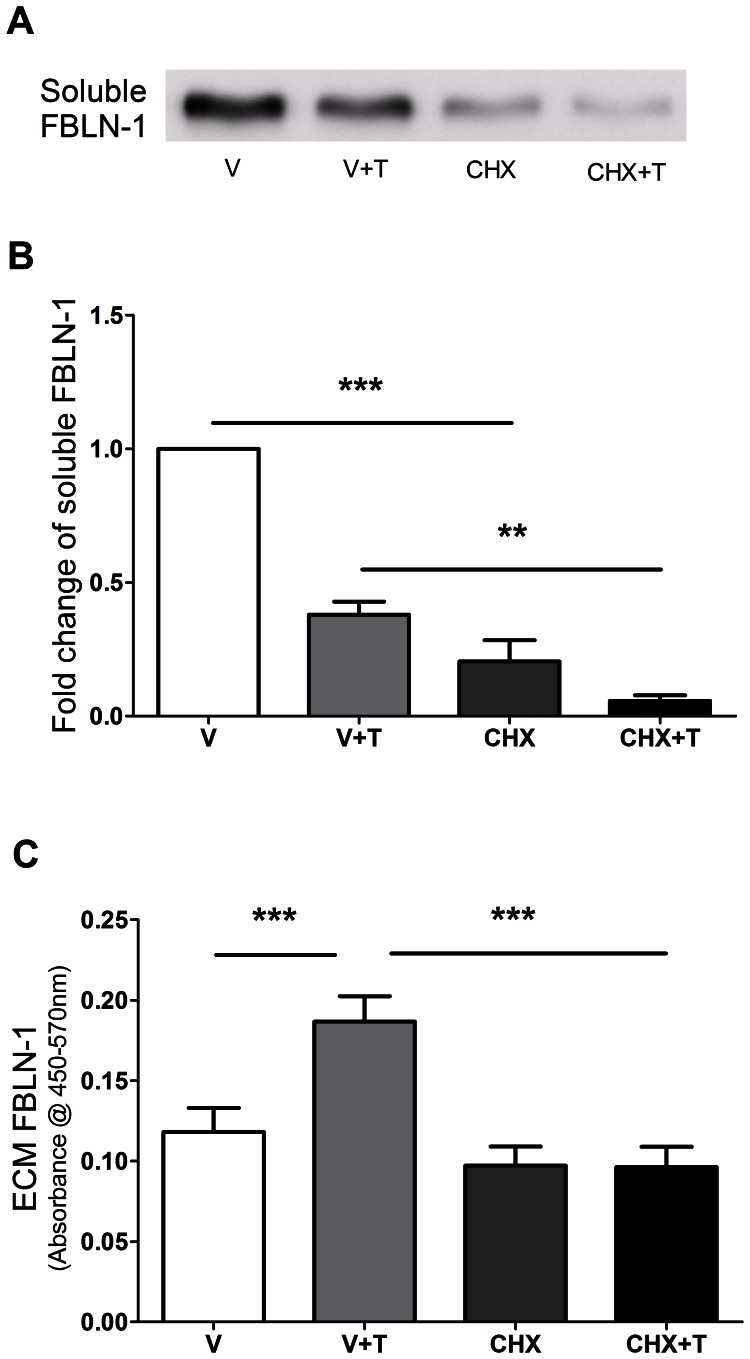
Cycloheximide inhibited soluble FBLN-1 synthesis and down-regulated the deposition of FBLN-1 induced by TGF-β_1_. After stimulation with 10 ng/ml TGF-β_1_ in the presence or absence of 0.5 µg/ml cycloheximide, the soluble FBLN-1 released from human ASM cells was detected by western blot (panel A). Data were normalized to cell number and results were expressed as the fold change compared with vehicle (panel B, n = 5). The deposition of FBLN-1 from human ASM cells in different treatments was measured by ECM ELISA and data were expressed as absorbance at 450 nm–570 nm (panel C, n = 5). Data were expressed as mean ± SEM and analysed by one-way ANOVA with Bonferroni’s multiple comparison test, **P<0.01, ***P<0.001. V: vehicle; CHX: cycloheximide; T: TGF-β_1_.

Human ASM cells were treated with 0.5 µg/ml cycloheximide for 1 hour before stimulation. After stimulation with or without 10 ng/ml TGF-β_1_ in the presence of cycloheximide for 72 hours, the amount of soluble FBLN-1 released from human ASM cells was significantly decreased by cycloheximide compared with vehicle control (p<0.001, p<0.01, n = 5) (Fig. 4A, 4B). Similarly, TGF-β_1_ induced deposition of FBLN-1 by human ASM cells also was significantly attenuated by cycloheximide (p<0.001, n = 5) (Fig. 4C). Soluble and ECM FN were significantly decreased by cycloheximide compared with vehicle control (soluble FN, p<0.01, n = 4, ECM FN, p<0.05, p<0.01, n = 5, Figs. S4A–C). Addition of cycloheximide did not have a significant effect on total cell number (Fig. S5).

### Soluble FBLN-1 is Incorporated into the ECM of Cycloheximide Treated Human ASM Cells

**Figure 5:**
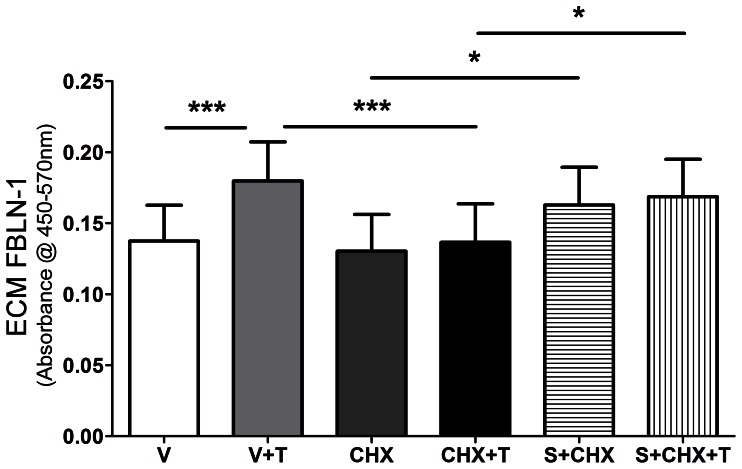
FBLN-1 from a soluble source is incorporated into the ECM. Cycloheximide treated and untreated human ASM cells were stimulated with or without 10 ng/ml TGF-β_1_ in quiescing medium or soluble FBLN-1 containing medium for 72 hours (n = 7). The incorporation of FBLN-1 into the matrix was detected by ECM ELISA and data were expressed as absorbance at 450 nm–570 nm. Data were expressed as mean ± SEM and analysed by one-way ANOVA with Bonferroni’s multiple comparison test, *P<0.05, ***P<0.001. V: vehicle; CHX: cycloheximide; S: soluble FBLN-1 containing medium; T: TGF-β_1_.

TGF-β_1_ decreased FBLN-1 mRNA and soluble FBLN-1 while increasing ECM FBLN-1. Cycloheximide decreased soluble FBLN-1 production and TGF-β_1_ induced ECM FBLN-1. Next we investigated if TGF-β_1_ induced ECM FBLN-1 could be derived from soluble FBLN-1 by adding exogenous source of FBLN-1 to human ASM cells which had been blocked from producing FBLN-1. Cycloheximide exposed ASM cells were treated with conditioned medium containing FBLN-1 with or without 10 ng/ml TGF-β_1_. After 72 hours, the incorporation of FBLN-1 into the ECM was significantly increased in the presence of the conditioned medium containing FBLN-1 (p<0.05, n = 7) (Fig. 5). However, the increased deposition of FBLN-1 was not different in the presence or absence of TGF-β_1_.

## Discussion

The architecture of the bronchial airway often undergoes prominent and permanent structural changes, including alterations of the molecular composition of the ECM. We hypothesized that ASM cells from people with COPD may constitutively produce higher levels of FBLN-1 than ASM cells from people without COPD, and TGF-β_1_ stimulation would further increase the production of FBLN-1. In the present study, we found TGF-β_1_ increased the deposition of ECM FBLN-1 protein by human ASM cells from both people with COPD and without COPD, whereas TGF-β_1_ decreased the soluble FBLN-1 from both COPD and non-COPD ASM cells. To confirm that the deposition of FBLN-1 was increased, the cellular and ECM FBLN-1 was measured and found to be increased in both COPD ASM cells and non-COPD ASM cells by TGF-β_1_. Next we assessed the regulation of FBLN-1, and found that TGF-β_1_ decreased FBLN-1 mRNA, and that FBLN-1 protein production was translationally controlled. Therefore we hypothesized that the increased ECM FBLN-1 following TGF-β_1_ stimulation was due to sequestration of soluble FBLN-1 and not de-novo synthesis. As there was not a commercially available source of FBLN-1 we exposed translationally inhibited ASM cells to conditioned medium containing FBLN-1 and found increased ECM FBLN-1.

We selected TGF-β_1_ as the stimulus to assess FBLN-1 production by human ASM cells because it is a potent inducer of ECM proteins, including FN and perlecan [19], [22], and is considered to play a significant role in fibrosis. In this study, we confirmed that TGF-β_1_ increased the expression of FN mRNA and the production of ECM and soluble FN by human ASM cells from people with COPD and without COPD.

Our previous studies found that TGF-β_1_ increased the deposition of FBLN-1 in asthmatic ASM cells only within 24 hours [13], and TGF-β_1_ increased the deposition of perlecan in COPD ASM cells only following 48 hours stimulation [22]. In this study, after stimulation up to 72 hours, there were no differences in the TGF-β_1_ induced FBLN-1 or FN between ASM cells from people with or without COPD. This suggests that the expression profiles of different ECM proteins, and their regulation is inherently different in cells from people with different diseases, however, we also have to consider the biological relevance of stimulation of cells with TGF-β_1_. As TGF-β_1_ is increased in COPD [15], [16] it is a reasonable assumption that cells in the COPD airways in-vivo are more likely to produce FBLN-1 in comparison to the cells from non-diseased airways, in which TGF-β_1_ level may not be raised.

To understand why ECM FBLN-1 was increased but soluble FBLN-1 was decreased by TGF-β_1_, we assessed the FBLN-1 mRNA in ASM cells after stimulation with TGF-β_1._ FBLN-1 mRNA was gradually down regulated by TGF-β_1_ over the time course of 72 hours. This reduction in FBLN-1 mRNA may be the cause of the decreased soluble FBLN-1 protein observed following TGF-β_1_ stimulation because of there was a reduction in the amount of FBLN-1 mRNA available for transcription. However, as the levels of FBLN-1 mRNA at the 4 h and 8 h time points was still quite stable, which also suggested that the possibility that the increased deposition of FBLN-1 was transcribed from the early time point FBLN-1 mRNA, and subsequently incorporated into the matrix.

It is still unknown whether TGF-β_1_ induced the deposition of FBLN-1 into the ECM directly through the activation of the cell surface receptors such as integrins, or if TGF-β_1_ increases other ECM proteins, which may bind to soluble FBLN-1 and anchor it in the ECM. In support of the possibility of integrin involvement, it is known that TGF-β_1_-increased FN deposition occurs via an integrin receptor on human ASM cells [19]. However, it is also known that FBLN-1 is not incorporated into the ECM by cells which fail to assemble FN [10], indicating that these two proteins have a critical interaction in other cell systems. We found that FN increased independently of increased synthesis of FBLN-1, but we do not know if FBLN-1 is the rate limiting step in FN incorporation into the ECM.

We used cycloheximide, which is an inhibitor of protein biosynthesis in eukaryotic organisms, to inhibit new protein synthesis. It exerts its effect by interfering with the translocation step in protein synthesis (movement of two tRNA molecules and mRNA in relation to the ribosome) thus blocking translational elongation. In this study, the cycloheximide treatment, at a concentration which was not cytotoxic, decreased the constitutive release of soluble FBLN-1 and inhibited TGF-β_1_ induced FBLN-1 deposition in the ECM. As expected, it also inhibited the production of soluble and deposited FN. We used soluble FBLN-1 containing medium to treat cycloheximide treated ASM cells in the presence or absence TGF-β_1_. We found that the addition of soluble FBLN-1 increased the deposition of FBLN-1 into the ECM even in the presence of cycloheximide. This increased deposition is unlikely to be the result of increased de-novo synthesis of other ECM proteins as the cells were treated with cycloheximide. However cellular processes independent of new protein translation would still occur, such as integrin activation, and these overall experiments indicate that soluble FBLN-1 influences the deposition of ECM FBLN-1. The increased deposition of FBLN-1 by TGF-β_1_ is likely due to incorporation of soluble FBLN-1 rather than de-novo synthesis. Furthermore as there was no difference in ECM FBLN-1 between cells treated with and without TGF-β_1_ in the presence of cycloheximide, this suggests that the increased deposition of FBLN-1 is not simply the result of TGF-β_1_ activated cell surface receptors.

In conclusion, this study demonstrates that TGF-β_1_ increased FBLN-1 independent of local de-novo synthesis. As FBLN-1 is increased in fibrotic tissues in a range of diseases and organs, this study suggests that potentially the FBLN-1 may be derived from cells or organs distant from those affected. Therefore soluble FBLN-1, as is found in serum, may potentially be a novel way of affecting the development and/or persistence of fibrosis in multiple organs.

## Supporting Information

Figure S1 TGF-β_1_ increased the deposited and cellular FN in human ASM cells. After 72 hours stimulation with TGF-β_1_, the deposition of FN by human ASM cells isolated from COPD (black bar, n = 12) and non-COPD (grey bar, n = 11) was measured by ELISA and data were expressed as absorbance at 450 nm–570 nm (panel A). Cellular FN and GAPDH from human ASM cells were detected by western blot (panel B). Data were normalized to GAPDH and expressed as fold change relative to control (n = 6, panel C). Data were expressed as mean ± SEM and analysed by two-way ANOVA with Bonferroni post tests, and paired t-test, *P<0.05, **P<0.01, compared with control.
(TIF)

Figure S2 TGF-β_1_ increased soluble FN from human ASM cells. Soluble FN released from human ASM cells was detected by western blot, following 72 hours stimulation with 10 ng/ml TGF-β_1_ (panel A). Data were expressed as fold change relative to control (n = 8, panel B). Data were expressed as mean ± SEM and analysed by paired t-test, *P<0.05, compared with control.
(TIF)

Figure S3 TGF-β_1_ increased FN gene expression in human ASM cells. COPD (black bar, n = 7) and non-COPD (grey bar, n = 5) human ASM cells were stimulated with 10 ng/ml TGF-β_1_, and FN mRNA expression was detected during the time course by real time PCR. Results were normalized to the endogenous control (18S rRNA), and expressed as fold change in FN mRNA compared with time matched control. Data were expressed as mean ± SEM and analysed by two-way ANOVA with Bonferroni post tests, **P<0.01, compared with time matched control.
(TIF)

Figure S4 Cycloheximide inhibited soluble FN synthesis and down-regulated the deposition of FN induced by TGF-β_1_. After stimulation with 10 ng/ml TGF-β_1_ in the presence or absence of 0.5 ug/ml Cycloheximide, the soluble FN released from human ASM cells detected by western blot (panel A). Data were normalized to cell number and results were expressed as the fold change compared with vehicle (panel B, n = 4). The deposition of FN from human ASM cells in different treatments was measured by ECM ELISA and data were expressed as absorbance at 450 nm–570 nm (panel C, n = 5). Data were expressed as mean ± SEM and analysed by one-way ANOVA with Bonferroni’s multiple comparison test, *P<0.05, **P<0.01, V: vehicle; CHX: cycloheximide; T: TGF-β_1_.
(TIF)

Figure S5 Cycloheximide had no effect on total cell number. After stimulation with 10 ng/ml TGF-β_1_ in the presence or absence of 0.5 ug/ml Cycloheximide, the cell number in each condition was counted (manual cell counts) (n = 5). Data were expressed as mean ± SEM and analysed by one-way ANOVA with Bonferroni’s multiple comparison test. V: vehicle; CHX: cycloheximide; T: TGF-β_1_.
(TIF)
