# Supplementary figures and images for: Differential Regulation of Extracellular Matrix and Soluble Fibulin-1 Levels by TGF-β1 in Airway Smooth Muscle Cells
Source: PLoS One. 2013 Jun 7;8(6):e65544. doi: 10.1371/journal.pone.0065544 (PMC3676355; doi:10.1371/journal.pone.0065544)

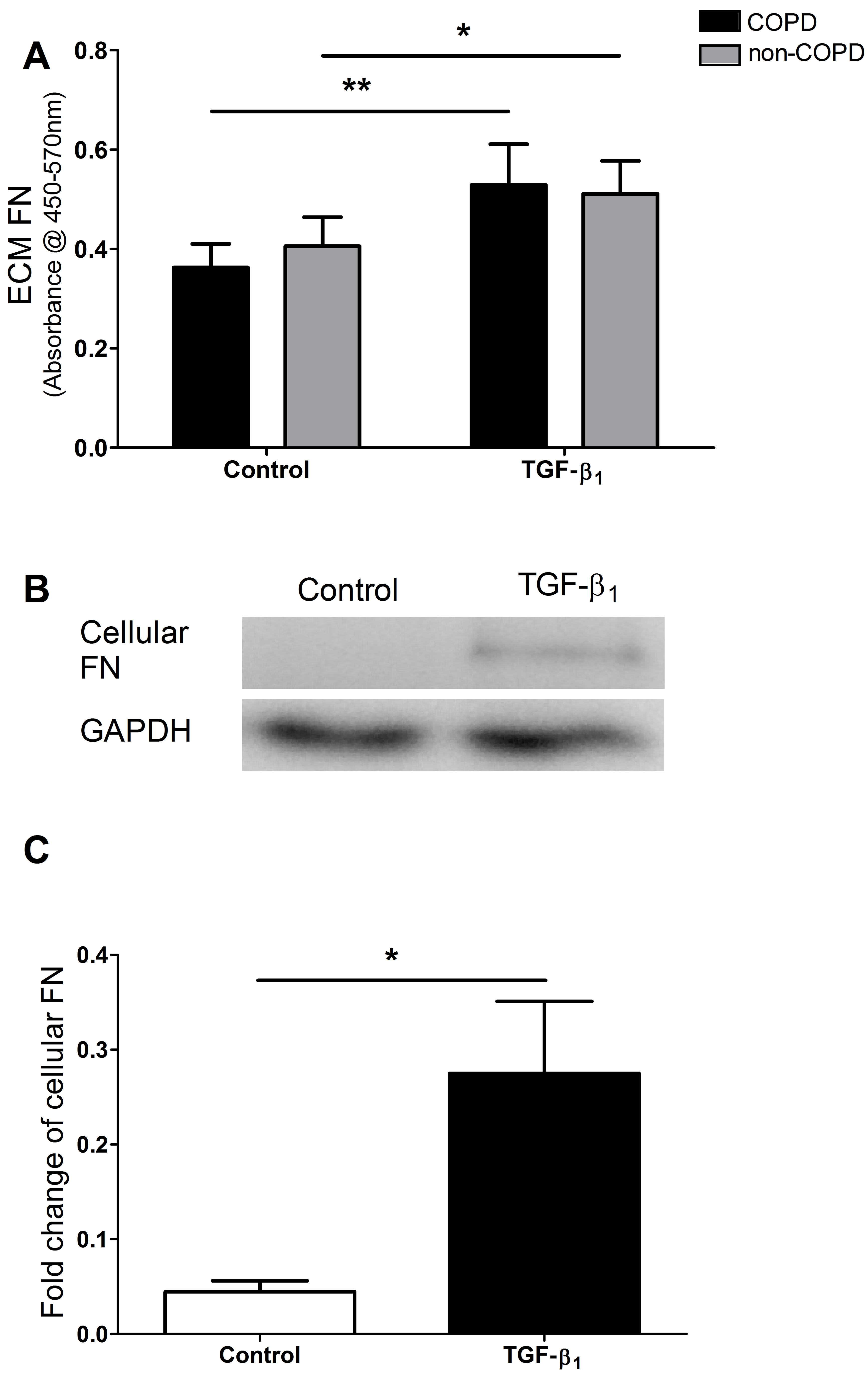

Supplement: Figure S1 — TGF-β1 increased the deposited and cellular FN in human ASM cells. After 72 hours stimulation with TGF-β1, the deposition of FN by human ASM cells isolated from COPD (black bar, n = 12) and non-COPD (grey bar, n = 11) was measured by ELISA and data were expressed as absorbance at 450 nm–570 nm (panel A). Cellular FN and GAPDH from human ASM cells were detected by western blot (panel B). Data were normalized to GAPDH and expressed as fold change relative to control (n = 6, panel C). Data were expressed as mean ± SEM and analysed by two-way ANOVA with Bonferroni post tests, and paired t-test, *P<0.05, **P<0.01, compared with control. (TIF) [file pone.0065544.s001.tif]

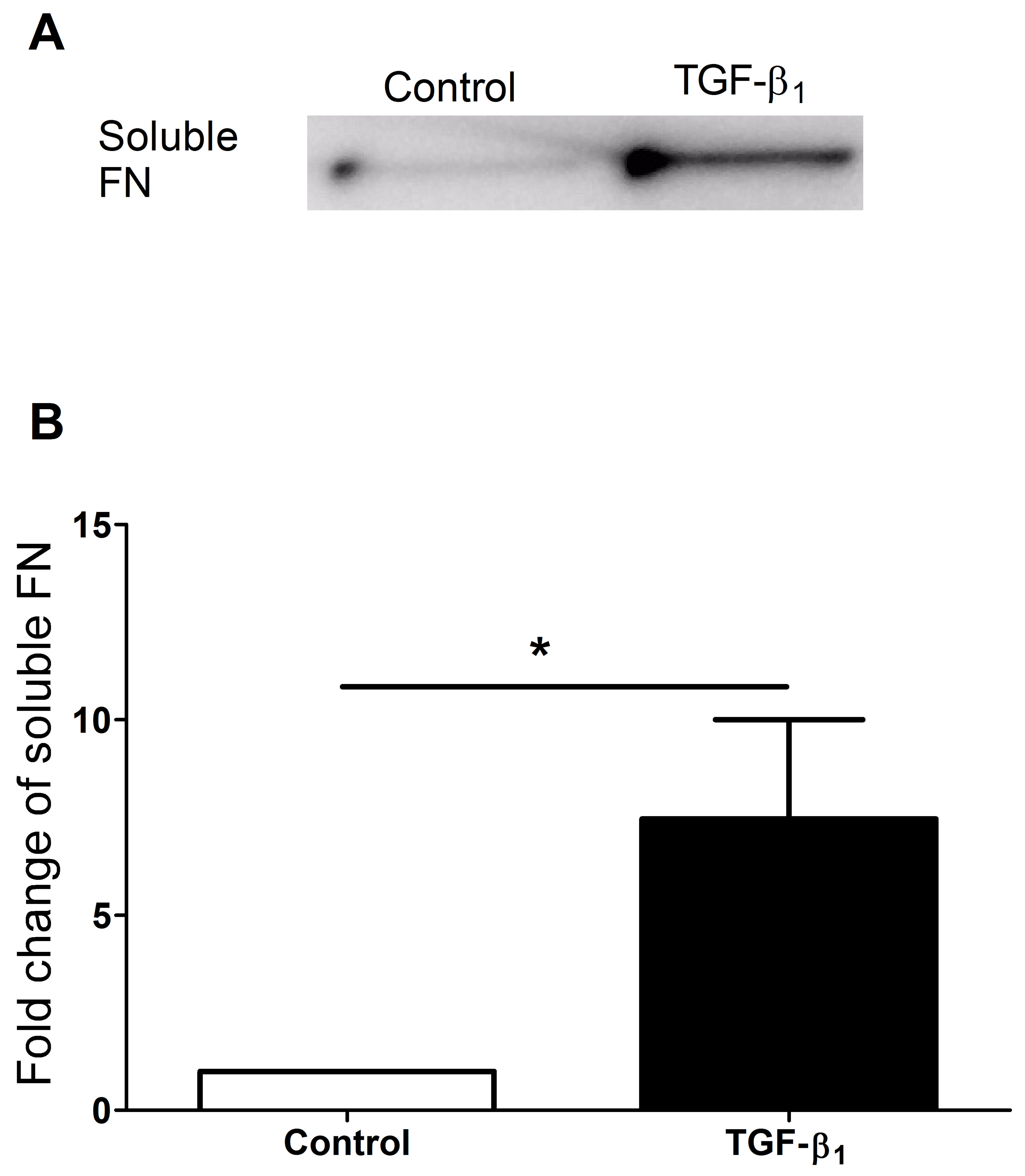

Supplement: Figure S2 — TGF-β1 increased soluble FN from human ASM cells. Soluble FN released from human ASM cells was detected by western blot, following 72 hours stimulation with 10 ng/ml TGF-β1 (panel A). Data were expressed as fold change relative to control (n = 8, panel B). Data were expressed as mean ± SEM and analysed by paired t-test, *P<0.05, compared with control. (TIF) [file pone.0065544.s002.tif]

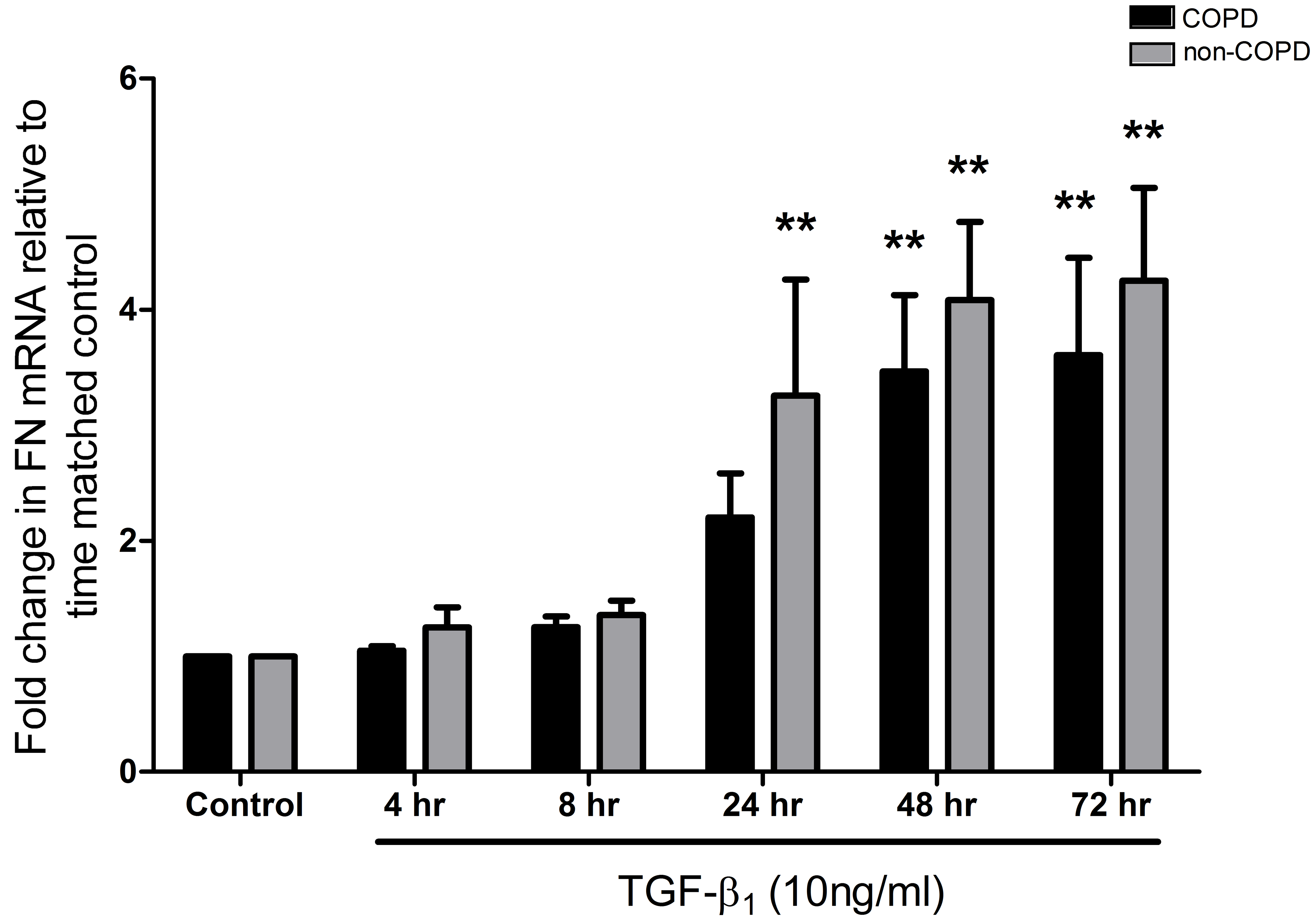

Supplement: Figure S3 — TGF-β1 increased FN gene expression in human ASM cells. COPD (black bar, n = 7) and non-COPD (grey bar, n = 5) human ASM cells were stimulated with 10 ng/ml TGF-β1, and FN mRNA expression was detected during the time course by real time PCR. Results were normalized to the endogenous control (18S rRNA), and expressed as fold change in FN mRNA compared with time matched control. Data were expressed as mean ± SEM and analysed by two-way ANOVA with Bonferroni post tests, **P<0.01, compared with time matched control. (TIF) [file pone.0065544.s003.tif]

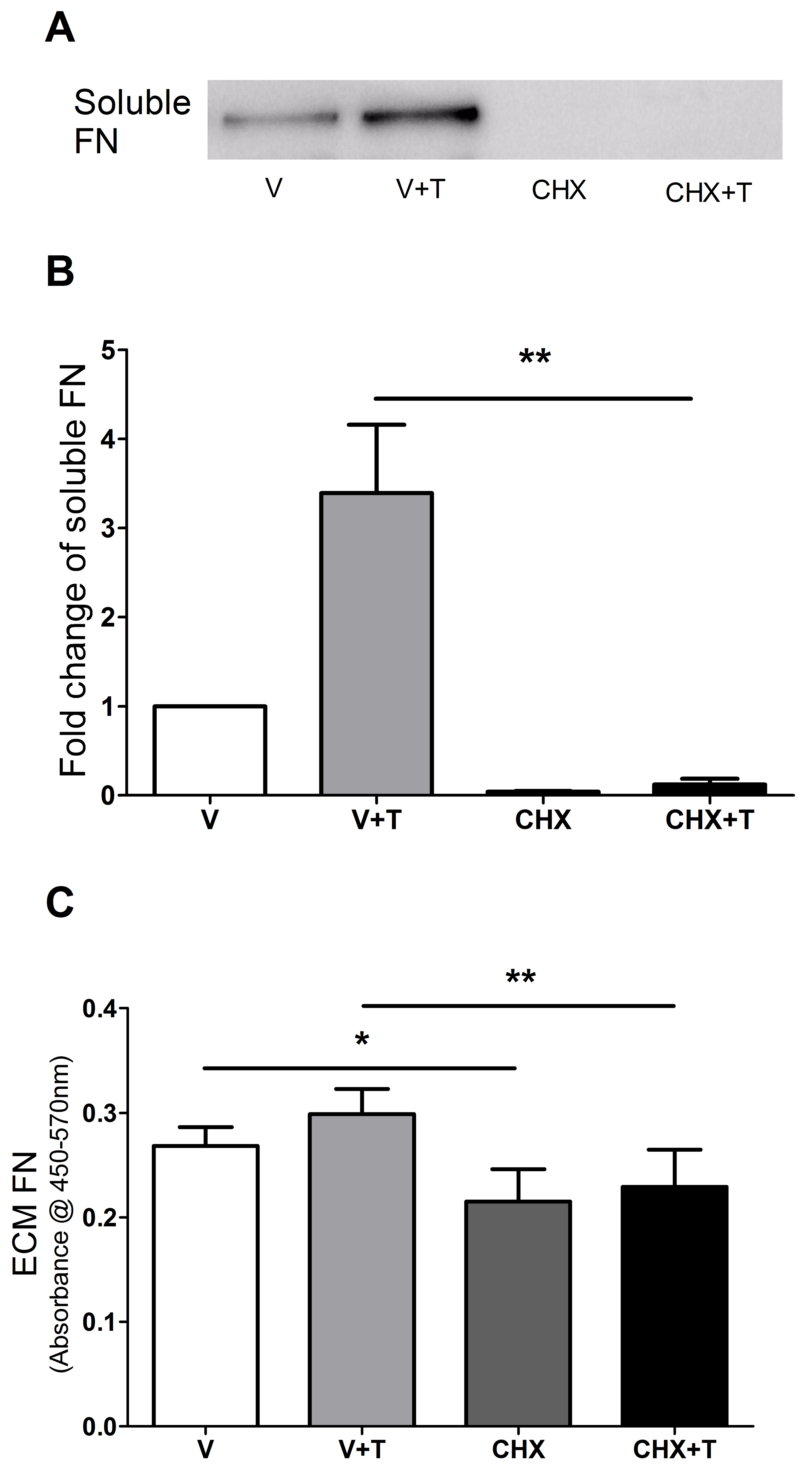

Supplement: Figure S4 — Cycloheximide inhibited soluble FN synthesis and down-regulated the deposition of FN induced by TGF-β1. After stimulation with 10 ng/ml TGF-β1 in the presence or absence of 0.5 ug/ml Cycloheximide, the soluble FN released from human ASM cells detected by western blot (panel A). Data were normalized to cell number and results were expressed as the fold change compared with vehicle (panel B, n = 4). The deposition of FN from human ASM cells in different treatments was measured by ECM ELISA and data were expressed as absorbance at 450 nm–570 nm (panel C, n = 5). Data were expressed as mean ± SEM and analysed by one-way ANOVA with Bonferroni’s multiple comparison test, *P<0.05, **P<0.01, V: vehicle; CHX: cycloheximide; T: TGF-β1. (TIF) [file pone.0065544.s004.tif]

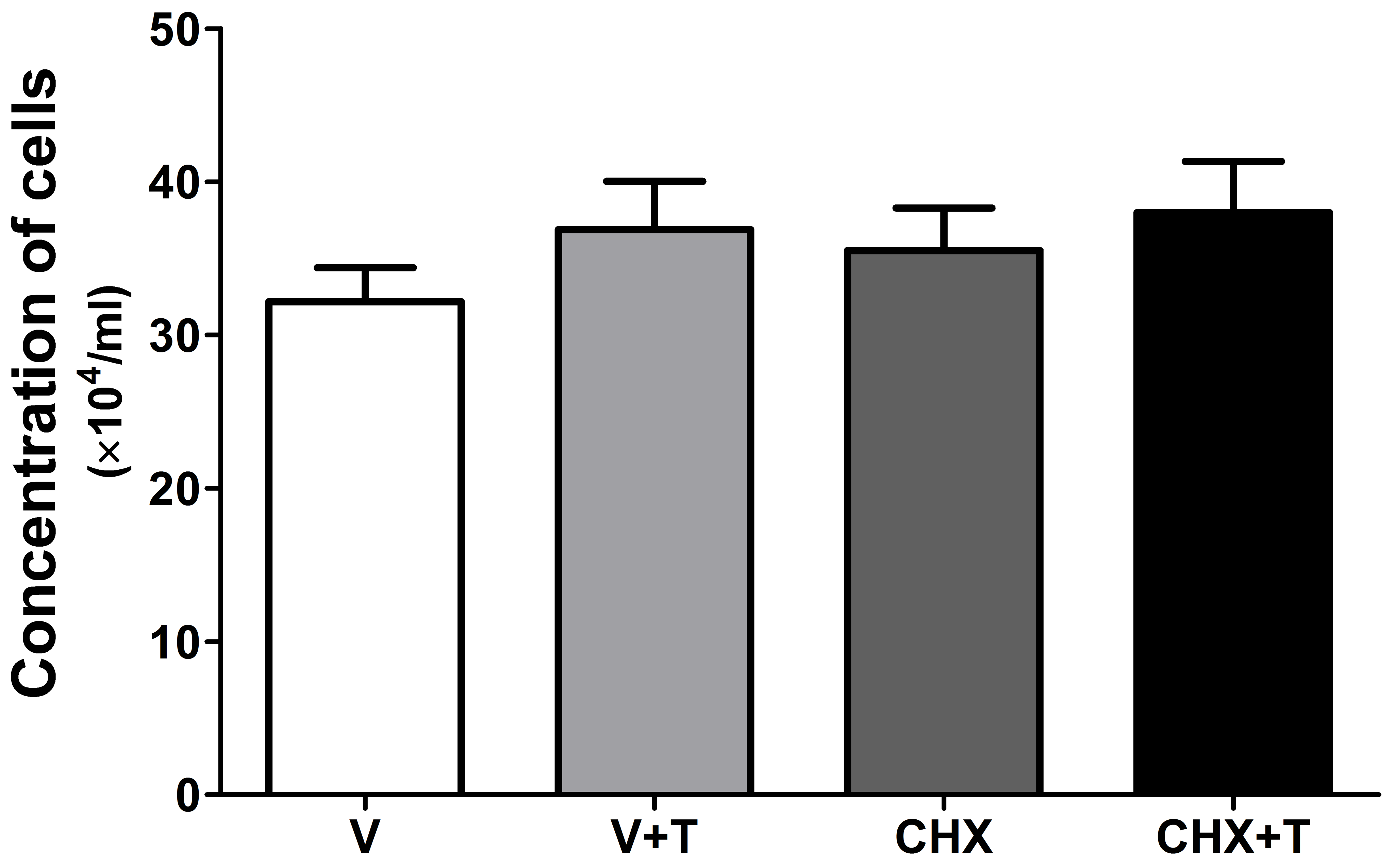

Supplement: Figure S5 — Cycloheximide had no effect on total cell number. After stimulation with 10 ng/ml TGF-β1 in the presence or absence of 0.5 ug/ml Cycloheximide, the cell number in each condition was counted (manual cell counts) (n = 5). Data were expressed as mean ± SEM and analysed by one-way ANOVA with Bonferroni’s multiple comparison test. V: vehicle; CHX: cycloheximide; T: TGF-β1. (TIF) [file pone.0065544.s005.tif]
